# Cemented mobile‐bearing medial unicompartmental knee arthroplasty provides long‐term implant survival and sustained functional performance in young and active patients aged 60 or below

**DOI:** 10.1002/ksa.12703

**Published:** 2025-05-26

**Authors:** Kevin‐Arno Koch, Michael Thapa, Johannes Weishorn, Mustafa Hariri, Benedict Lotz, Kevin Knappe, Tobias Reiner, Tilman Walker

**Affiliations:** ^1^ Department of Orthopaedic Surgery University Hospital of Heidelberg Heidelberg Germany

**Keywords:** clinical research, knee osteoarthritis, patient reported outcome measure, treatment outcome, unicompartmental knee arthroplasty, young adults

## Abstract

**Purpose:**

To evaluate long‐term outcomes of cemented, mobile‐bearing medial unicompartmental knee arthroplasty (UKA) in patients aged 60 or younger, focusing on implant survival, functional results, patient‐reported outcome measures (PROMs), and radiographic findings over >10 years.

**Methods:**

This retrospective single‐centre study included 119 knees (102 patients). Implant survival was evaluated using Kaplan–Meier analysis. PROMs included the Oxford Knee Score (OKS), American Knee Society Score (AKSS), UCLA Activity Score and Visual Analogue Scale (VAS). Assessments were performed preoperatively, at mid‐term (2–10 years), and at long‐term follow‐up (>10 years). OKS and AKSS were analysed in relation to Patient Acceptable Symptom State (PASS) thresholds. Radiographs were graded using the Kellgren–Lawrence classification to evaluate lateral osteoarthritis (LOA) progression.

**Results:**

The implant survival rate was 86.7% (95% CI: 78.5–91.9%) at 15 years, and 81.7% (95% CI: 71.4%–88.5%) at 17.5 years. Revision surgery was required in 18 knees (mean time to revision: 8.7 years), primarily due to progression of LOA. All PROMs improved significantly from baseline to the final follow‐up (mean: 16 years; *p* < 0.0001). Although minor functional declines occurred between mid‐ and long‐term follow‐ups, these were not statistically significant (*p* > 0.05), except for functional AKSS. At the final assessment, 96% of patients exceeded the PASS threshold for OKS, 84% for AKSS objective, and 80% for AKSS functional scores. Radiographic LOA progression was frequent but did not significantly impair functional outcomes.

**Conclusions:**

Cemented mobile‐bearing medial UKA in patients aged ≤60 years demonstrated high long‐term implant survival and sustained functional benefit. Even in the presence of radiographic LOA progression, clinical outcomes remained excellent. UKA represents a durable and effective treatment for younger patients with isolated medial compartment osteoarthritis.

**Level of Evidence:**

Level IV.

**Abbreviations**
AKSS‐F: functional American Knee Society Score
AKSS‐O: objective American Knee Society Score
BMI: body mass index
CI: confidence interval
CR: cruciate‐retaining
FU: follow‐up
KLS: Kellgren–Lawrence score
MCID: minimal clinically important difference
OKS: oxford knee score
PASS: patient acceptable symptom state
PRO(M): patient‐reported outcome (measures)
PS: posteriorstabilised
ROM: range of motion
SC: semi‐constrained
SD: standard deviation
TKA: total knee arthroplasty
UCLA: University Of California Los Angeles activity score
UKA: unicompartmental knee arthroplasty
VAS: visual analogue scale

## INTRODUCTION

Primary joint replacement rates are projected to rise significantly, not only among the elderly, but also in younger patients [15]. Unicompartmental knee arthroplasty (UKA) has seen increasing implantation rates in recent years, particularly in younger patients, according to national database reports [11, 40]. Despite its effectiveness in treating severe anteromedial osteoarthritis, UKA remains underutilised, especially among younger individuals [10, 17]. UKA offers distinct advantages over total knee arthroplasty (TKA), including higher patient satisfaction, faster recovery, improved range of motion and more physiological knee kinematics [3, 4, 42, 44]—all of which are particularly important to active younger patients [17, 41]. However, UKA is associated with higher revision rates, particularly in younger patients who are considered at greater risk [13, 25, 38].

Studies on UKA in younger patients (<60 years) have shown promising results, with outcomes comparable to those of older populations [10, 18]. Recent reports indicate excellent mid‐ to long‐term survivorship, with 96%–98% survival at 5 years [14, 19, 35] and 81%–96% at 10 years [9, 13, 18, 23, 43] in patients under 60 years of age. Furthermore, functional outcomes remain very good to excellent at 10 years postoperatively [13, 18, 43], though there is evidence of gradual decline over time [31]. However, data on UKA outcomes beyond 10 years, particularly in younger, high‐demand patients, remains scarce.

Therefore, this study aims to evaluate the long‐term clinical, radiographic, and implant survival outcomes of cemented medial mobile‐bearing UKA in patients aged 60 or younger at the time of surgery. We hypothesise that implant survival in young patients will demonstrate long‐term results (>10 years) comparable to existing literature, with sustained high functional outcomes.

## PATIENTS AND METHODS

### Study cohort and follow‐up

This retrospective single‐centre study reviewed the first consecutive 122 medial UKA in 105 patients aged 60 or younger at the time of surgery who had undergone mobile‐bearing cemented medial UKA between January 2001 and October 2007. Following the exclusion of three patients (three knees) who withdrew consent, 119 knees (102 patients) comprised the study cohort. Mid‐term results of this cohort have been reported previously and were available for the present study [35]. Baseline characteristics and demographics are presented in Table 1.

**Table 1:** Patient demographics and perioperative patient‐related factors.

Total numbers of patients/knees	102/119
Primary diagnosis	
Osteoarthritis (%)	116 (97.5%)
Osteonecrosis (%)	3 (2.5%)
Sex	
Men (%)	60 (50.4%)
Women (%)	59 (49.6%)
Operated side	
Right (%)	55 (46.2%)
Left (%)	64 (53.8%)
Mean age at time of surgery in years (range)	54 (35–60)
Mean body mass index (BMI) (range)	31.4 (20.3–57.7)

The study was conducted in accordance with the Helsinki Declaration of 1975, as revised in 2013 [1], and were approved by the institutional review Ethics Commission of the Medical Centre, University of Heidelberg (S‐594/2022). Informed consent was obtained from all participating patients prior to enrolment in this study.

Clinical and radiographic follow‐up examinations were recommended at regular intervals after 3 months, 1 year, 3 years, 5 years and every 5 years thereafter following the index surgery. For the present study, patient's assessment was conducted from October 2022 to June 2023 and patients were explicitly invited for regular examinations. Consequently, patients were deemed eligible for a minimum 15‐year follow‐up. In the event that the aforementioned examination had already been regularly attended by a patient, or that they were unable to attend the examination due to health or other reasons, they were contacted via post or telephone in order to provide additional information on complications or revision procedures, and to complete the patient‐reported outcome measure (PROM) questionnaires. For deceased patients, information between the last clinical follow‐up and death was obtained using information from relatives, general practitioners, and hospital records.

**Figure 1:**
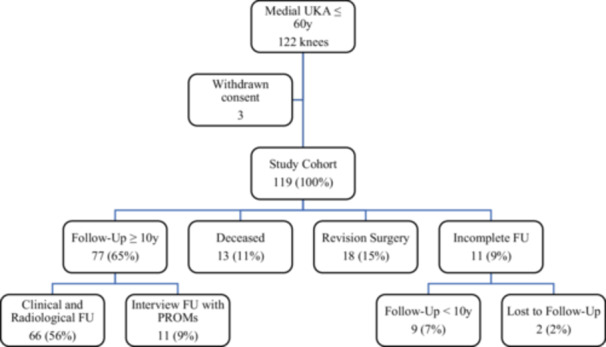
Flowchart visualising patient allocation at the most recent follow‐up.

At the most recent follow‐up, twelve patients (13 knees) had died from unrelated causes, with the implants remaining intact and no revision surgery required. Seventeen patients (18 knees) underwent revision procedures. Of the 88 knees available for follow‐up, 77 had at least 10 years of follow‐up, with 71 exceeding 15 years. Nine patients (11 knees) had incomplete follow‐up: eight patients (nine knees) had follow‐up shorter than 10 years (range: 2.8–8.0 years), and one patient was lost to follow‐up within the first year. The median follow‐up for the entire cohort was 15.7 years. A patient flow chart is presented in Figure 1.

### Indication and surgical treatment

As previously described [35, 43], medial unicompartmental knee arthroplasty (UKA) was performed for advanced osteoarthritis of the medial compartment (116 knees) or avascular necrosis of the medial femoral condyle (three knees), with intact ACL and collateral ligaments. Varus deformity was manually reducible, and there was no lateral compartment osteoarthritis on valgus stress radiographs. Lateral osteophytes or mild joint narrowing did not preclude surgery. Exclusion criteria included deep eburnation, bone grooving on the medial patellar facet, rheumatoid arthritis, fixed varus deformity, or flexion deformity >15°. All procedures used the mobile‐bearing Oxford partial knee implant (Zimmer Biomet Inc., Warsaw, Indiana, USA) with a minimally invasive, cemented approach by 12 surgeons. Full weight‐bearing was permitted postoperatively.

### Clinical and radiographic review

A comprehensive data set was collected, including demographics, complications, revisions, and patient‐reported outcome measures (PROMs) preoperatively, at 2–10 years [35], and at >10 years (first and second follow‐ups). PROMs included the American Knee Society scores (AKSS‐O and AKSS‐F), Oxford Knee Score (OKS), UCLA activity score, and Visual Analogue Scale (VAS). A recently published Patient Acceptable Symptomatic State (PASS) for AKSS and OKS was used to categorise the scores [36]. Kaplan–Meier analysis was used to assess long‐term implant survival, with ‘revision for any reason’ (removal or replacement of at least one component) as the endpoint. Patient‐related factors influencing long‐term implant survival were also examined.

For the radiological review, only patients who had radiological follow‐up for ≥10 years without revision surgery were included. Two independent orthopaedic surgeons (KK, TW) performed the radiographic assessments, using standardised radiographs aligned with fluoroscopic control to ensure views parallel to the tibial component in the AP view and the femoral component in the lateral view. The analysis focused on signs of implant loosening, subsidence, radiolucent lines, and osteoarthritis progression in the lateral compartment. Kellgren–Lawrence scores (KLS) were used to grade osteoarthritis, with both preoperative and recent radiographs considered. Interobserver reliability for the KLS grading was calculated. Additionally, functional outcomes and range of motion were compared between patients with none/mild (KLS ≤ 2) and moderate/severe (KLS ≥ 3) lateral knee osteoarthritis.

### Statistical analysis

Data were recorded and analysed using Microsoft Excel (Microsoft Corporation, Redmond, Washington, USA), SPSS Version 27.0 (IBM SPSS Statistics, IBM, Armonk, NY, USA) and GraphPad Prism Version 10.0 (GraphPad Software, San Diego, CA), and G‐Power 3.1 (Heinrich Heine University, Düsseldorf, Germany). The level of significance was set at *p* < 0.05.

Descriptive data and scores were calculated as absolute frequencies and means with standard deviations, with the exception of the UCLA as median and range. Normality of continuous variables was assessed using the Shapiro–Wilk test. Non‐parametric Mann–Whitney *U* tests were used for group comparisons.

Implant survival was assessed using the Kaplan–Meier estimator with 95% confidence intervals. Censoring was applied for patients with incomplete follow‐up including the loss of one patient or deceased due to unrelated causes, ensuring accurate survival estimation. Based on the sample size, survival was calculated up to 17.5 years, with a minimum of 24 knees still being at risk.

A Cox proportional hazards regression model was used for uni‐ and multivariate analysis, including age at operation and BMI as continuous independent variables as well as sex as a categorical variable.

The interobserver reliability of the radiographic assessment was calculated and reported using Cohen's Kappa, which resulted in substantial agreement, with an interclass correlation of 0.66.

A post hoc power analysis was performed to determine the validity of the results. With a small effect size of *ω* = 0,3, an available number of patients of *n* = 81 and an alpha of 0.05, the calculated statistical power to detect an underlying difference in clinical outcome is 85%. However, to detect differences in radiographic analysis with adequate power, a post hoc power analysis indicated that a larger group size would be required.

## RESULTS

### Survival analysis

Revision surgery was required in 18 knees following an average of 8.7 years, primarily due to the progression of osteoarthritis in the lateral compartment (seven knees). Detailed information on revision surgery is provided in Table 2.

**Table 2:** Characteristics of patients requiring revision surgery.

Revision number	Time to revision (years)	Age	Reason for revision	Procedure	Implant type
01	0.1	60	Suspected early infection	Bearing exchange	UKA
02	0.8	60	Unexplained pain	Revision to TKA	CR
03	1.1	54	Unexplained pain	Bearing exchange	UKA
04	1.9	60	Impingement	Revision to TKA	CR
05	3.4	59	Aseptic loosening	Revision to TKA	SR
06	4.7	62	Progression of lateral osteoarthritis	Revision to TKA	CR
07	6.5	67	Bearing breakage	Bearing exchange	UKA
08	7.8	58	Aseptic loosening	Revision to TKA	SR
09	7.9	68	Bearing dislocation	Bearing exchange	UKA
10	9.4	63	Progression of lateral osteoarthritis	Revision to TKA	CR
11	9.5	62	Progression of lateral osteoarthritis	Revision to TKA	CR
12	10.0	62	Wear	Revision to TKA	SR
13	11.1	69	Periprosthetic fracture	Revision to TKA	DFR
14	14.5	65	Progression of lateral osteoarthritis	Revision to TKA	CR
15	15.4	68	Progression of lateral osteoarthritis	Revision to TKA	CR
16	16.1	69	Bearing breakage	Revision to TKA	PS
17	16.7	59	Progression of lateral osteoarthritis	Revision to TKA	n.a.
18	20.0	78	Progression of lateral osteoarthritis	Revision to TKA	n.a.

Abbreviations: CR, cruciate‐retaining; DFR, distal femoral replacement; n.a., not available; PS, posterior‐stabilised; SC, semi‐constrained; TKA, total knee arthroplasty; UKA, unicompartmental knee arthroplasty.

**Figure 2:**
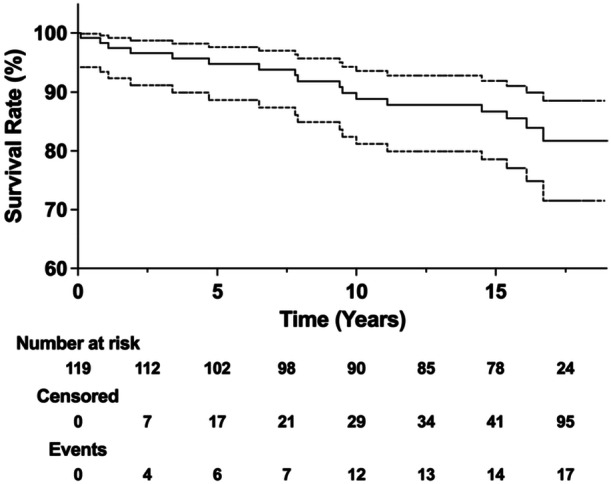
The implant survival rate of revision surgery for any reason estimated with the Kaplan–Meier analysis was 86.7% (95% CI: 78.5%–91.9%; 78 knees at risk) at 15 years and 81.7% (95% CI: 71.4%–88.5%; 24 knees at risk) at 17.5 years. respectively.

The Kaplan–Meier survival analysis with revision for any reason as the endpoint yielded an implant survival rate of 94.8% (95% CI: 88.7%–97.6%; 102 knees at risk) at 5 years, 89.8% (95% CI: 82.3%–94.2%; 90 knees at risk) at 10 years, 86.7% (95% CI: 78.5%–91.9%; 78 knees at risk) at 15 years and 81.7% (95% CI: 71.4%–88.5%; 24 knees at risk) at 17.5 years (see Figure 2).

Univariate Cox regression analysis revealed no significant impact of gender, age (≤50 vs. >50), or BMI (≤30 vs. >30, ≤40 vs. >40) on the risk of revision surgery. Similarly, multivariate analysis confirmed that these factors did not significantly confound the outcomes.

### Functional outcome

A total of 81 knees were available for functional evaluation with a minimum follow‐up of 10 years. Of these, four patients underwent functional evaluation over a period of 10 years postoperatively prior to undergoing subsequent revision surgery after an interval of several years. Mean follow‐up was 16 years. Patients showed significant improvement in pain, function and range of motion compared to preoperative values (*p* < 0.0001). The mid‐ to long‐term scores between the first (2–10 years) and second (>10 years) follow‐ups decreased slightly over time (Table 3) but did not reach significance (*p* > 0.05), except for AKSS‐F (*p* < 0.0001).

**Table 3:** Patient‐reported outcome measures (PROMs) and range of motion (ROM) preoperatively, at first follow‐up (2–10 years) and second follow‐up (>10 years).

	Preoperatively (*n* = 119)	1. FU 2–10 y (*n* = 109)	2. FU > 10 y (*n* = 81)
OKS	25.65 (6.88)	41.31 (6.94)	39.60 (8.48)
AKSS‐O	46.27 (14.59)	89.25 (13.89)	87.85 (14.49)
AKSS‐F	58.30 (19.19)	90.47 (13.74)	80.38 (20.01)
UCLA	3 (1–9)	7 (2–10)	6 (2–10)
VAS	7.63 (2.28)	1.60 (2.45)	1.74 (2.27)
ROM	112 (16.83)	128 (13.88)	123 (13.16)

*Note*: Mean (standard deviation) for American Knee Society Scores (AKSS‐O and AKSS‐F), Oxford Knee Score (OKS), Visual Analogue Scale (VAS), Range of Movement (ROM); median (range) for California Los Angeles activity score (UCLA).

At the latest follow‐up, the AKSS‐O score exceeded the PASS in 84% of cases, the AKSS‐F score in 80%, and the OKS score in 96%. According to the developers of the PROMs, 82% of the patients had an excellent or good outcome based on the OKS (OKS > 29).

### Radiographic outcome

A total of 66 knees were evaluated radiographically, with a minimum follow‐up of 10 years and a mean follow‐up of 15 years. No evidence of implant loosening was observed in these knees. Physiological radiolucency under the tibial component was seen in 11 knees (16.7%).

Preoperatively, 27.3% of patients showed Grade I osteoarthritis in the lateral compartment, and 1.5% had Grade II osteoarthritis. No patients had osteoarthritis greater than Grade II. At final follow‐up, 36.4% of patients had Grade I osteoarthritis, 24.2% had Grade II, 12.1% had Grade III, and 1.5% had Grade IV. In total, 59.1% of knees demonstrated progression of lateral osteoarthritis, with 27.3% showing a progression of more than one grade.

At 15 years, patients with moderate to severe lateral knee osteoarthritis (KLS ≥ 3) had outcomes similar to those with none to mild osteoarthritis (KLS ≤ 2) (*p* > 0.05; Table 4). However, range of motion was significantly better in patients with none to mild osteoarthritis (*p* = 0.039).

**Table 4:** Patient‐reported outcome measures (PROMs) and ROM by lateral osteoarthritis severity.

	KLS ≤ 2 (*n* = 57)	KLS ≥ 3 (*n* = 9)	*p* value
OKS	38.30 (9.22)	42.00 (3.02)	0.767
AKSS‐O	89.04 (14.09)	86.50 (10.30)	0.113
AKSS‐F	79.55 (21.43)	80.63 (15.45)	0.844
UCLA	6 (2–10)	6 (3–7)	0.942
VAS	1.80 (2.29)	1.88 (2.1)	0.880
ROM	124 (13.06)	116 (11.78)	**0.039**

*Note*: Mean (standard deviation) for American Knee Society Scores (AKSS‐O and AKSS‐F), Oxford Knee Score (OKS), Visual Analogue Scale (VAS), Range of Movement (ROM); median (range) for California Los Angeles activity score (UCLA); KLS, Kellgren–Lawrence Score.

## DISCUSSION

The most important finding of the present study was that cemented mobile‐bearing medial unicompartmental knee arthroplasty (UKA) in active patients aged ≤60 years achieved high satisfactory long‐term outcomes, with a 17.5‐year implant survival rate of 81.7%, sustained high functional scores, low pain levels, and consistently high patient satisfaction. Although radiographic progression of lateral compartment osteoarthritis was frequently observed at a mean follow‐up of 15 years, this did not correlate with poorer clinical outcomes.

Joint replacement in younger patients remains challenging due to a higher lifetime revision risk [13, 38]. Nonetheless, several studies have reported comparable survivorship between patients below and above 55 or 60 years of age [10, 18, 32]. Greco et al. and Walker et al. reported a 10‐year survival rate of 86% in patients under 50 years following Oxford UKA [9, 43], whereas Mohammad et al. reported a lower rate of 81.4% in patients aged ≤60 years [23]. The majority of studies, however, indicate survival rates exceeding 90% at 10 years, which aligns with the findings of the present investigation.

Long‐term outcomes beyond 15 years remain sparsely documented and show considerable variability. Designer studies report survival rates of approximately 91% at 15 years [28], while non‐designer series show more heterogeneous results, ranging from 76.4% to 90.6% [7, 20, 27].

Despite the inclusion of a younger patient population—typically associated with increased revision rates—the 15‐year survival rate of 86.7% observed in the present study compares favourably with previous cohorts. No other study to date has reported a mean survival beyond 15 years for medial UKA in patients aged 60 years or younger.

Comparisons with total knee arthroplasty (TKA) in younger patients suggest similar long‐term implant survival in some reports [29, 30]. Perdisa et al. demonstrated a 15‐year survival of 78.7% for patients under 50 and 89.4% for those aged 50–65 years [30]. However, registry‐based data typically favour TKA over UKA in terms of implant survival rates [8, 29], though these comparisons should be interpreted with caution due to confounding factors such as unmatched cohorts, broader indications, and variability in surgeon experience [6]. Matched cohort analyses, including those from the National Joint Registry (NJR), show smaller differences between procedures [25]. Therefore, surgical volume, case selection, and revision thresholds are crucial factors affecting UKA outcomes [16, 24].

Progression of lateral compartment osteoarthritis emerged as the primary reason for revision in the current cohort, particularly in mid‐ to long‐term follow‐up. This failure mode has been frequently reported in the literature [26, 37]. Aseptic loosening was observed in only two cases, suggesting a lower incidence than in other studies [26, 37, 39]. Revision for unexplained pain was as common as aseptic loosening. Unexplained pain is a significant cause of failure in the first five years after surgery [37]. Although some authors attribute unexplained pain to unrecognised osteoarthritis in other compartments [39], this could not be substantiated in the present study. In the absence of radiographic progression, the indication for revision surgery due to non‐specific symptoms should be cautiously considered, as outcomes may not improve [2].

Superior functional outcomes, quicker recovery, and greater patient satisfaction have been consistently reported following UKA when compared to TKA [3, 44]. The results of the present study confirm long‐term maintenance of excellent PROMs, with high AKSS and OKS scores persisting up to a mean of 16 years postoperatively. Previous studies have shown comparable values at 10 years, with mean OKS values ≥ 38 and AKSS‐O scores of 80–90 [20, 26, 28]. Distinctive outcomes have also been demonstrated for younger cohorts with regard to PROMs [10, 18]. Hamilton et al. reported similarly favourable outcomes in patients under 60, with an OKS of 41, AKSS‐O of 81 and AKSS‐F of 87 at 10 years [10].

The present study is the first to demonstrate excellent long‐term functional scores, with high AKSS and OKS scores, in a young and active cohort with an average follow‐up period of 16 years. Furthermore, the high level of functional results was able to be sustained over this period of time. This observation of maintenance has also been documented in other studies [5, 20, 28], albeit with shorter follow‐up periods. Although minor declines in function were observed at the latest follow‐up, these remained below minimal clinically important difference (MCID) thresholds (approximately 5 points for OKS [12] and 7.2 points for AKSS‐O [21]) and thus were not considered clinically relevant.

PASS thresholds provide valuable insights into perceived treatment success. At final follow‐up, 84% of patients surpassed the PASS threshold for AKSS‐O, 80% for AKSS‐F, and 96% for OKS [36]. Considering that PROMs tend to decline with increasing follow‐up time [34, 36], these results reflect a sustained patient‐centred benefit.

Radiographic progression of lateral osteoarthritis was detected in 59.1% of cases, although only 13.6% exhibited advanced changes at a mean of 15 years. This aligns with previous reports indicating limited progression in most cases [22, 33]. For instance, Searle et al. found moderate to severe lateral osteoarthritis in 9.8% of 163 patients at 15 years [33]. Despite a trend toward lower PROMs in patients with advanced lateral osteoarthritis, functional outcomes remained in a good range and did not significantly differ from those without progression. Consequently, radiological findings alone should not dictate the indication for revision, particularly in asymptomatic or mildly symptomatic individuals.

The present findings provide valuable guidance for clinical decision‐making in younger patients with isolated medial compartment osteoarthritis. The long‐term durability of medial UKA, combined with high patient‐reported satisfaction and function, supports its use as a reliable treatment option in selected cases. These insights can assist surgeons in counselling patients, setting realistic expectations, and optimising implant choice in routine clinical practice.

### Limitations and strengths

This retrospective study is subject to inherent limitations, including the potential for selection and recall bias, as well as incomplete data acquisition. The absence of a control group limits direct comparison with other treatment strategies. The relatively small sample size, especially in comparison with registry‐based studies, may affect the generalisability of the findings. Moreover, procedures were performed by multiple surgeons in a university setting, potentially introducing variability in surgical technique and outcomes.

Despite these limitations, the study provides robust long‐term data, with follow‐up of at least 15 years in over 80% of surviving non‐revised patients. The inclusion of a well‐defined, active cohort aged ≤60 years at the time of surgery fills a relevant gap in the current literature. Additionally, the availability of mid‐term results enabled longitudinal assessment of outcome trajectories, and the detailed radiographic evaluation offers insights into the clinical relevance of progression of lateral osteoarthritis—an aspect often missed in similar studies.

## CONCLUSIONS

The present study demonstrated a high long‐term implant survival rate for medial UKA in patients aged 60 or younger, with 87% survival at 15 years and 82% at 17.5 years. Additionally, excellent functional outcomes, as measured by PROMs, were well maintained over an average follow‐up of 16 years. Although progression of lateral osteoarthritis was common in non‐revised patients, it did not impair functional outcomes.

## AUTHOR CONTRIBUTIONS

*Conceptualisation and methodology*: Kevin‐Arno Koch and Tilman Walker. *Investigation*: Kevin‐Arno Koch, Michael Thapa, Johannes Weishorn, Mustafa Hariri, and Benedict Lotz. *Formal analysis and visualisation*: Kevin‐Arno Koch and Michael Thapa. *Writing–original draft preparation*: Kevin‐Arno Koch. *Writing–review and editing*: Johannes Weishorn, Mustafa Hariri, Benedict Lotz, Kevin Knappe, Tobias Reiner, Tilman Walker. *Supervision*: Kevin‐Arno Koch and Tilman Walker.

## CONFLICT OF INTEREST STATEMENT

The authors declare no conflicts of interest.

## ETHICS STATEMENT

The current study was approved by the Ethics Commission of the Medical Center, University of Heidelberg (S‐594/2022). Written informed consent was obtained of every patient before inclusion.

## Data Availability

The data will be available upon reasonable request.
